# Calcium signaling is involved in diverse cellular processes in fungi

**DOI:** 10.1080/21501203.2020.1785962

**Published:** 2020-07-14

**Authors:** Avishek Roy, Ajeet Kumar, Darshana Baruah, Ranjan Tamuli

**Affiliations:** Department of Biosciences and Bioengineering, Indian Institute of Technology Guwahati, Guwahati, India

**Keywords:** Calcium signalling, calcineurin-Crz1 pathway, Ca^2+^ transporter, calmodulin and NCS-1, fungi, phospholipase

## Abstract

Calcium (Ca^2+^) is a universal signalling molecule of life. The Ca^2+^ signalling is an evolutionarily conserved process from prokaryotes to eukaryotes. Ca^2+^ at high concentration is deleterious to the cell; therefore, cell maintains a low resting level of intracellular free Ca^2+^ concentration ([Ca^2+^]_c_). The resting [Ca^2+^]_c_ is tightly regulated, and a transient increase of the [Ca^2+^]_c_ initiates a signalling cascade in the cell. Ca^2+^ signalling plays an essential role in various processes, including growth, development, reproduction, tolerance to stress conditions, and virulence in fungi. In this review, we describe the evolutionary aspects of Ca^2+^ signalling and cell functions of major Ca^2+^ signalling proteins in different fungi.

## Evolution of calcium as a unique signalling molecule

Since the evolution of life on earth, where water covers three-fourth of the surface, both calcium (Ca^2+^) and magnesium (Mg^2+^) have evolved as divalent cations with a difference in the ability to form a complex with water molecules. Ca^2+^ has formed when oxygen and neon fused with successive particles (Clapham 2007). Ca^2+^ has a high degree of hydration and can accommodate 6–8 water molecules (Williams 2006). Ca^2+^ binds less tightly to water than Mg^2+^ and can precipitate phosphate, which may be lethal to the cell (Clapham 2007). Ca^2+^ can easily interact with molecules of complex geometry like proteins due to its unique properties such as charge, ionic radius, polarisability, and hydration energy (Brini et al. 2013a, 2013b). Therefore, cell efficiently controls the Ca^2+^ level for its survival and signalling. Thus, Ca^2+^ has evolved as a universal signalling molecule, signifying its importance in the evolution of life that started about 3.5 billion years ago (Plattner and Verkhratsky 2013). From the time life evolved, ATP has emerged as the central molecule, which was responsible for the formation of DNA/RNA subsequently (Ponnamperuma et al. 1963; Galimov 2009); and ATP synthesis was dependent on low Ca^2+^ concentration (Verkhratsky and Parpura 2014). It is also possible that in the process of evolution, Ca^2+^ assisted in the stability of DNA or RNA molecules, which exist as primitive stable molecules for the evolution of life (Jaiswal 2001). Since the early days of bacteria and protozoan evolution, Ca^2+^ was considered as a molecule of cell signalling, much before it got established as a ubiquitous secondary messenger molecule in the eukaryotic system (Shemarova and Nesterov 2005; Case et al. 2007), and the evolution of proteins allows the messenger function (Williams 2006). Thus, Ca^2+^ has evolved as an essential signaling ion across the different forms of life (Plattner and Verkhratsky 2013).  Due to its versatile role in cell signaling, Ca^2+^ is also  considered as a molecule for life and death (Berridge 1998; Berridge et al. 1998).

## Ca^2+^ concentration gradient is the main switch behind the Ca^2+^ signalling machinery

**Figure 1.**
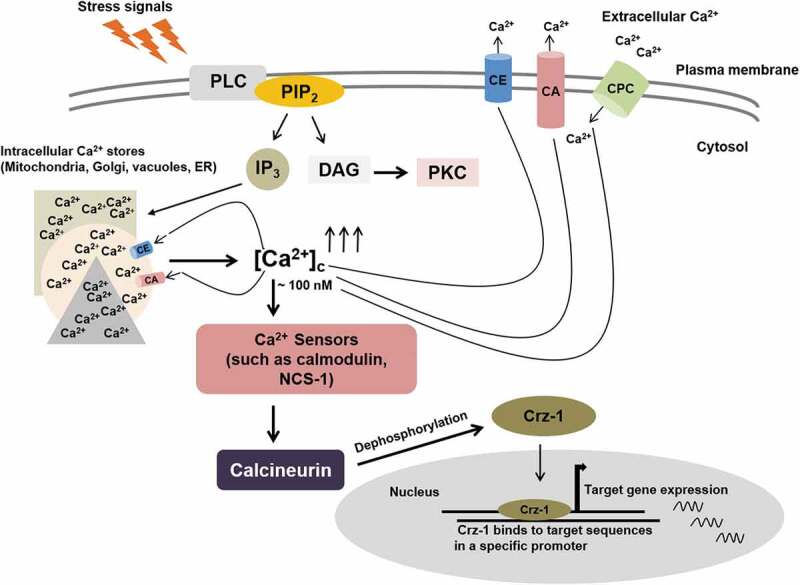
Overview of calcium signalling machinery in fungi. The membrane-bound phosphoinositide-specific phospholipase C (PLC) hydrolyzes phosphatidylinositol-4, 5-bisphosphate (PIP_2_) to produce two important second messenger molecules inositol 1,4,5-triphosphates (IP_3_) and diacylglycerol (DAG). IP_3_ induces Ca^2+^ release from intracellular stores, including mitochondria, Golgi, vacuoles, and endoplasmic reticulum (ER). DAG activates protein kinase C (PKC) that is involved in various signalling processes (Clapham 2007). PLCs also response to different stress signals (Barman et al. 2018). The resting intracellular free Ca^2+^ concentration ([Ca^2+^]_c_) is about 100 nM, an increase (shown using arrows pointing upward) of the [Ca^2+^]_c_ is detected by various Ca^2+^ sensing proteins such as calmodulin (CaM) and NCS-1, which activate specific downstream signalling cascade. Calcineurin-Crz-1 pathway is shown here as an instance of a downstream signalling cascade. Ca^2+^ and CaM activate the serine/threonine phosphatase calcineurin that dephosphorylates the transcription factor Crz-1 for its nuclear location and subsequent expression of target genes in responses to stimuli. Excess Ca^2+^ is removed from the cytosol by Ca^2+^ exchangers (CE) and Ca^2+^ -ATPases (CA) proteins, whereas Ca^2+^ permeable channels (CPC) are required for the influx of extracellular Ca^2+^; and these processes are required to maintain Ca^2+^ homoeostasis in the cell (Tamuli et al. 2013)

The Ca^2+^ concentration outside the cell is as high as 10^−3^ M (Chin and Means 2000), the cytosolic free Ca^2+^ concentration ([Ca^2+^]_c_) at resting state is maintained at ∼100 nM; therefore, cells maintain a more than 10,000-fold gradient across the plasma membrane (Berridge et al. 2003). Cells store excess Ca^2+^ in various intracellular stores, including endoplasmic reticulum (ER), mitochondria, and vacuoles (Cornelius and Nakashima 1987). In the endoplasmic reticulum, the Ca^2+^ concentration ([Ca^2+^]_ER_) is maintained at several hundred µM. Besides, cells also need to maintain intracellular Ca^2+^ homoeostasis to avoid severe Ca^2+^ fluctuations and their effects (Berridge et al. 2003). Specific receptors and channels mediate the entry of Ca^2+^ across the plasma membrane in response to stimuli, including membrane depolarisation, mechanical stretch, and external agonists. The inositol 1,4,5-triphosphates receptors (IP_3_R) and the ryanodine receptors (RyR) induces Ca^2+^ release from the internal stores (Mikoshiba and Hattori 2000; Zeng et al. 2003; Hamilton 2005). Several proteins, including the plasma membrane Ca^2+^-ATPase (PMCA), the sarcoplasmic/endoplasmic reticulum Ca^2+^-ATPase (SERCA), the Na^+^/Ca^2+^ exchanger, and the mitochondrial uniporter are responsible for sequestering the excess Ca^2+^ from the cytosol by transporting Ca^2+^ either to external medium or into different cellular compartments (Berridge et al. 2003). Thus, in response to various stimuli, the Ca^2+^ signalling pathway is activated and causes expression of the specific target genes in the nucleus (Figure 1). In this review, we briefly describe major families of Ca^2+^ signalling proteins (Table 1) and their roles in cellular processes in different fungi.

**Table 1.** Calcium signalling genes in different yeast and fungal species

Organism	Genome Size (Mb)	Number of protein coding genes	Number of Calcium signaling genes	Subfamily	References
*Saccharomyces cerevisiae*	12.1	5, 596	40	Ca^2+^ permeable channel (3), Ca^2+^/cation ATPases (5), Ca^2+^ exchanger (4), phospholipase C (2), CaM (1), Ca^2+^/CaM regulated proteins (25)	Zelter et al. 2004; Otero et al. 2010; Engel et al. 2014
*Schizosaccharomyces pombe*	13.8	4,940	18	Ca^2+^ permeable channel (3), CaM (2), Ca^2+^/cation ATPases (8), Phospholipase (1), Ca^2+^/CaM regulated proteins (4)	Takeda and Yamamoto 1987; Toda et al. 1993; Yoshida et al. 1994; Okorokova-Facanha et al. 2003; Wood et al. 2002; Cortés et al. 2004
*Aspergillus fumigatus*	29.4	9,926	13	Ca^2+^ permeable channel (3), Ca^2+^ exchanger (3), Ca^2+^/cation ATPases (3), Phospholipase (3), CaM (1)	Birch et al. 1996; Nierman et al. 2005; Dinamarco et al. 2012; de Castro et al. 2014
*Beauveria bassiana*	33.7	10,366	21	Ca^2+^ permeable channel (3), Ca^2+^/cation ATPases (6), Ca^2+^ exchanger (5), CaM (1), Ca^2+^/CaMK (2)Ca^2+^/CaM regulated proteins (4)	Xiao et al. 2012; Fan et al. 2012; Li et al. 2015
*Candida albicans*	14	6,100	11	Ca^2+^/cation ATPases (1), Ca^2+^ permeable channel (4), CaMK (1), CaM (1), Ca^2+^/H^+^ exchanger (1), Phospholipase (3)	Bennett et al. 1998; Andaluz et al. 2001; Jones et al. 2004; Luna-Tapia et al. 2019
*Cryptococcus neoformans*	19	6,572	40	Ca^2+^ permeable channel (3), Ca^2+^/cation ATPases (6), Ca^2+^ exchanger (3), phospholipase C (2), CaM (1), Ca^2+^/CaM regulated proteins (21), Ca^2+^/CaMK (4)	Odom et al. 1997; Kmetzsch et al. 2011; Bahn and Jung 2013; Lee et al. 2016
*Fusarium oxysporum*	59.9	17,735	4	Ca^2+^ Permeable channel (3), Phospholipase (1)	Ma et al. 2010; Su et al. 2017
*Magnaporthe grisea*	40.3	11,109	42	Ca^2+^ Permeable channel (3), Ca^2+^/cation ATPases (12), Ca^2+^ exchanger (6), phospholipase C (4), CaM (1), Ca^2+^/CaM regulated proteins (16)	Zelter et al. 2004; Dean et al. 2005
*Neurospora crassa*	40	10,082	48	Ca^2+^ Permeable channel (3), Ca^2+^/cation ATPases (9), Ca^2+^ exchanger (8), phospholipase C (4), CaM (1), Ca^2+^/CaM regulated proteins (23)	Galagan et al. 2003; Zelter et al. 2004; Tamuli et al. 2013

## Phospholipases and their significance in Ca^2+^ signalling pathway in fungi

Phospholipases are a diverse class of enzymes involved in hydrolysing membrane phospholipids, mainly glycerophospholipids (Köhler et al. 2006; Hong et al. 2016). Phospholipases act on membrane phospholipids to produce small lipophilic signalling molecules like free fatty acids (FFAs), diacylglycerol (DAG), phosphatidic acid (PA), and lysophospholipids (LPLs) (Köhler et al. 2006; Hong et al. 2016). Phospholipases are of two types, acyl hydrolases, and phosphodiesterases. Phospholipases are classified, based on the specific ester linkage they cleave within phospholipid molecule, into four broad classes such as phospholipase A (PLA_1_ and PLA_2_), phospholipase B (PLB), phospholipase C (PLC), and phospholipase D (PLD) (Köhler et al. 2006). Both PLA and PLB are acyl hydrolases, while PLC and PLD belong to the phosphodiesterase class of phospholipases (Richmond and Smith 2011). The membrane-bound phosphoinositide-specific PLC cleaves phosphatidylinositol-4, 5-bisphosphate (PIP_2_) into two important second messengers, inositol 1,4,5-triphosphates (IP_3_) that induces Ca^2+^ release from internal stores and diacylglycerol (DAG) that activates protein kinase C (PKC), and triggers a range of cellular activities (Cornelius and Nakashima 1987; Chae et al. 2007; Clapham 2007). The IP_3_ causes release of Ca^2+^ to activate calmodulin (CaM)-dependent enzymes (Berridge 1993), IP_3_ also acts as the precursor of many inositol polyphosphates like IP_5_ or IP_6_ (York et al. 1999). Majority of the eukaryotic PLCs contain five conserved domains, comprising of two catalytic domains X and Y, an N-terminal pleckstrin homology (PH) domain for interaction with the membrane phospholipid, a C-terminal Ca^2+^ dependent C2 domain for binding to phospholipids, and an EF-hand motif for Ca^2+^ binding and interaction of the PH domain with phospholipid (Sutton et al. 1995; Yamamoto et al. 1999). PLC is important for various cellular processes and pathogenicity in several organisms, including filamentous fungi. The budding yeast *Saccharomyces cerevisiae* contains Plc1p, a phosphatidylinositol-specific phospholipase C (PI-PLC), which shows sequence homology to mammalian PI-PLC-δ isoforms (Flick and Thorner 1993). The Plc1p is necessary for growth at nonpermissive temperatures (above 35°C), survival under osmotic stress, and utilisation of alternative carbon sources like galactose, raffinose, or glycerol at permissive temperatures (23 to 30°C) (Flick and Thorner 1993). In the fission yeast *Schizosaccharomyces pombe, plc1-1* is a homolog of *plc1*, mutation of *plc1-1* induced by nitrosoguanidine caused sensitivity to high amounts of phosphate in the medium

(Fankhauser et al. 1995). The growth defect of the *plc1-1* deletion mutant was partially restored in low inositol and low phosphate minimal media containing a high concentration of nitrogen, which suggests a potential role of *plc1-1* in ammonium sensing (Fankhauser et al. 1995). In the grey mould fungus *Botrytis cinerea, Plc1* homolog *BcPLC1* is required for vegetative growth, conidial formation, germination, and virulence (Schumacher et al. 2008). In the human-pathogenic fungus *Candida albicans*, the *PLC1* homolog *CaPLC1* is an essential gene and its conditional mutant showed increased sensitivity to a high concentration of sorbitol or NaCl, increased sensitivity to lower (18°C) or higher temperatures (43°C), and reduced growth in medium containing galactose, but not glucose, as the sole carbon source (Kunze et al. 2005). The *CaPLC1* conditional mutant was also sensitive to nocodazole that inhibits chromosome segregation, and showed reduced growth in filamentous growth-inducing conditions and on media containing only arginine as the sole nitrogen source (Kunze et al. 2005). However, two additional *PLC* genes in *CaPLC2* and *CaPLC3* genes were non-essential for growth as loss of *Caplc2* and *Caplc3* did not show any visible growth defects in *C. albicans* (Knechtle et al. 2005; Kunze et al. 2005). Moreover, in a mouse systemic infection model, the *CaPLC2* and *CaPLC3* mutants showed survival rate similar to the wild type, suggesting that both *CaPLC2* and *CaPLC3* are not essential for virulence (Knechtle et al. 2005; Kunze et al. 2005). In the citrus fungal pathogen, *Alternaria alternata*, the *PLC1* homolog is important for vegetative growth, conidial formation, Ca^2+^ homoeostasis, and virulence (Tsai and Chung 2014). In encapsulated yeast and human pathogen *Cryptococcus neoformans, CnPlc1*, a homolog of the mammalian PI-PLC-*δ* is essential for cellular homoeostasis and virulence (Lev et al. 2013). The model filamentous fungus *Neurospora crassa* possesses four novel PLC-δ proteins, including PLC-1, which is highly divergent among the natural isolates (Galagan et al. 2003; Borkovich et al. 2004; Gavric et al. 2007; Barman and Tamuli 2015, 2017; Barman et al. 2018). The *plc-1* mutant, generated using repeat-induced point mutation (RIP; Selker et al. 1987), is viable, but showed reduced growth, abnormal hyphal morphology, lower turgor, increased sensitivity to low extracellular and increase intracellular Ca^2+^ concentrations, and responded differently to PLC inhibitor 3-nitrocoumarin (Gavric et al. 2007). In another study, phenotypes of the RIP-generated and knockout mutants of *plc-1* were different, and analysis of the mutant phenotypes suggested a role for *plc-1* in hyphal tip growth in *N. crassa* (Lew et al. 2015). Moreover, the *plc-1* knock out mutant displays aberrant hyphal morphology in the presence of Ca^2+^ ionophore A23187, accumulates an increased amount of carotenoid, and shows reduced survival during oxidative and thermal stress (Barman and Tamuli 2015; Barman et al. 2018). In the rice-blast fungus *Magnaporthe oryzae*, PI-PLC-δ isoform *MoPLC1* regulates intracellular Ca^2+^ fluxes and plays an essential role in fungal development, appressorium formation, and pathogenicity (Rho et al. 2009).

## Ca^2+^ transporters and their involvement in cell processes in fungi

In fungi, six major types of Ca^2+^ transporters, including Ca^2+^ pumps, Ca^2+^/H^+^ exchangers (Lange and Peiter 2020), high-affinity calcium system (HACS), low-affinity calcium system (LACS), TRP-like Ca^2+^ channels, and mitochondrial Ca^2+^ uniporter (MCU) have been identified (Tisi et al. 2016). The HACS and LACS are two critical Ca^2+^ uptake systems that are conserved across different fungi and mediate the entry of Ca^2+^ under different cellular conditions (Martin et al. 2011). In fungi, Ca^2+^ release from internal stores such as Golgi bodies, endoplasmic reticulum, vacuole, and mitochondria are carried out by P-type Ca^2+^ ATPases driving the energy from the synthesis of ATP to transfer Ca^2+^ against the ion gradient (Li et al. 2019). In *A. fumigatus*, McuA is a Ca^2+^ uniporter localised to the mitochondrial membrane, and the deletion of *mcuA* results in disruption of the mitochondrial Ca^2+^ homoeostasis, suggesting its role in Ca^2+^ uptake (Song et al. 2016). In addition, the deletion of *mcuA* also causes resistance to azoles and oxidative stress; however, overexpression of *mcuA* restores the azole sensitivity phenotype in a deletion mutant of *agcA* that encodes for a mitochondrial carrier protein in *A. fumigatus* (Song et al. 2016). The knockouts of *cchA, midA*, and *yvcA*, which are the homologues of the *S. cerevisiae* genes encoding for voltage-gated *CCH1*, stretch-activated *MID1*, and vacuolar *YVC1* Ca^2+^ channels, respectively, were not virulent in mice model of invasive aspergillosis, suggesting that these transporters contribute to virulence in *A. fumigatus* (de Castro et al. 2014). In fungi, vacuoles are one of the major Ca^2+^ stores, the Ca^2+^ ATPases and the Ca^2+^/H^+^ exchangers are important transporters that guide the entry of Ca^2+^ into the vacuoles (Pittman 2011). The *pmcA, pmcB*, and *pmcC* type Ca^2+^ transporters in *A. fumigatus* are homologues of the *S. cerevisiae* plasma membrane Ca^2+^-ATPase *PMC1* (Cunningham and Fink 1994), transcribed by the calcineurin A-CrzA pathway, and necessary for growth and survival under the Ca^2+^ stress condition (Dinamarco et al. 2012). In *A. fumigatus*, the *pmcA* conditional mutant was not virulent in the mice model of invasive aspergillosis, suggesting that *pmcA* has a role in conferring virulence and pathogenicity to the fungi; *pmcA* also regulates the metabolism of Ca^2+^ and Mn^2+^ (Dinamarco et al. 2012). There are different classes of P-type Ca^2+^ ATPases such as plasma membrane Ca^2+^-ATPase (PMCA), sarco (endo) plasmic reticulum Ca^2+^-ATPase (SERCA) and secretory pathway Ca^2+^-ATPase (SPCA) are ATP-driven pumps involved in the active transport and homoeostasis of Ca^2+^ (Sze et al. 2000; Carafoli 2002; Ton and Rao 2004). In *S. cerevisiae*, deletion of the Ca^2+^-ATPase Pmr1p results in the lack of adequate amount of Ca^2+^ within the Golgi, an increase in the cytosolic Ca^2+^ concentration causing vacuolar fragmentation possibly to sequester excess Ca^2+^ (Kellermayer et al. 2003). In *S. pombe*, Cps5p is the Pmr1p homolog, which plays a role in the cell wall formation, protein glycosylation, and maintains intracellular Ca^2+^ homoeostasis by depletion of the excessive cytosolic Ca^2+^ via the interaction with a vacuolar Ca^2+^-ATPase homolog, Pmc1p (Cortés et al. 2004). In *C.albicans*, the deletion of a Ca^2+^-ATPase gene *CaPMR1*, the homolog of *PMR1* in *S. cerevisiae*, results in altered glycosylation, causing a defect in the cell wall formation and loss of virulence (Bates et al. 2005). In *N. crassa*, the deletion of a PMCA family of Ca^2+^ ATPase NCA-2 causes restricted growth, sensitivity to increasing concentration of Ca^2+^ in the media, and female sterile phenotype (Bowman et al. 2011; Deka and Tamuli 2013). In addition, the *nca-2* gene plays a role in carotenoid accumulation, regulation of the circadian clock with a Ca^2+^ sensor *ncs-1*, and thermotolerance with a cation-ATPase *trm-9* in *N. crassa* (Deka and Tamuli 2013; Laxmi and Tamuli 2015).

## Calmodulin is a major calcium-binding protein involved diverse cell functions in fungi

The change in the Ca^2+^ concentration inside the cell is detected by an array of Ca^2+^ sensors belonging to the EF-hand family of proteins. CaM is a primary Ca^2+^ sensor containing EF-hand (Lewit-Bentley and Réty 2000) motifs, and conserved from lower to higher eukaryotes (Chin and Means 2000). Because of its evolutionary conservation, CaM transduces about 300 effectors for the activation of the downstream signalling cascade in the eukaryotes (Halling et al. 2016). The *S. cerevisiae* CaM has three functional EF-hands; disruption of this gene has a lethal phenotype, and therefore, essential for viability (Davis et al. 1986). In *S. pombe*, disruption of *cam1* causes growth defects and abolishes cell division (Takeda and Yamamoto 1987). The *cam1* disruption results in improper chromosomal segregation, hyper condensation, uneven distribution of the chromosomal material, and absence of the spindle body proteins responsible for spore formation in *S. pombe* (Itadani et al. 2010). Immunofluorescence assay of the GFP-tagged *cam1* protein in *S. pombe* indicated the localisation of the Cam1 to the spindle body fibres and also to the sites responsible for polarised cell growth (Moser et al. 1997; Itadani et al. 2010). Calmodulin also mediates protein phosphorylation via calmodulin-dependent kinase in the presence of *N*-acetyl-D-glucosamine (GlcNAc), which is involved in germ tube formation causing morphogenesis of *C. albicans*; however, calmodulin inhibitor trifluoperazine (TFP) inhibits the phosphorylation and germ tube formation (Roy and Datta 1987; Paranjape et al. 1990). In addition, calmodulin binds to an integral membrane protein Dfi1p that activates mitogen-activated protein kinase (MAPK) Cek1p, which is required for invasive filamentation in *C. albicans* (Davis et al. 2013). In *C. neoformans, CAM1* is required for the infestation in the human body at 37°C (Kraus et al. 2005). In the presence of calcineurin inhibitor FK506, the temperature-sensitive *cam1-ts* mutant of *C. neoformans* showed defects in growth and bud formation at 25°C (Kraus et al. 2005). The *cam1-ts* mutant also showed reduced *CAM1* expression and growth defect at 37°C, which was not complemented when transformed with a calmodulin independent calcineurin A allele (*CNA1-AI*Δ) lacking the coding region for the C-terminal calmodulin-binding site and the autoinhibitory domain (Kraus et al. 2005). Therefore, *CAM1* has a role in both calcineurin-dependent and independent developmental pathways in *C. neoformans* (Kraus et al. 2005). In *M. grisea*, CaM is required for early-stage appressorium formation and conidial germination (Lee and Lee 1998; Liu and Kolattukudy 1999). The *cmd* is an essential gene in *N. crassa*; therefore, knockout of the *cmd* gene is not viable (Tamuli et al. 2013). CaM antagonists trifluoperazine (TFP) and chlorpromazine (CPZ) caused defects in hyphae formation, reduced growth, and impaired sexual development in *N. crassa* (Laxmi and Tamuli 2015). In addition, the RIP-generated *cmd* mutants showed reduced growth, less carotenoid accumulation, decreased survival in exposure to ultraviolet (UV) irradiation, and defect in the sexual development causing female sterility (Laxmi and Tamuli 2017). CaM also interacts with Ca^2+^/CaM-dependent kinases, including Ca^2+^/CaMK-2 that is required for full fertility in *N. crassa* (Kumar and Tamuli 2014; Laxmi and Tamuli 2017).

## Neuronal calcium sensor-1 regulates growth, sexual development, and Ca^2+^ homoeostasis in fungi

Neuronal calcium sensor-1 (NCS-1), which belongs to the family of EF-hand containing protein, binds to Ca^2+^ and senses the change in the concentration of the Ca^2+^ inside the cell (Burgoyne 2007; Tamuli et al. 2011). The NCS-1 was identified as frequenin (Frq1), enriched in the synapses of the *Drosophila melanogaster* nervous system, which facilitates the frequency-dependent release of neurotransmitter (Pongs et al. 1993). Additionally, Frq2, paralogue of Frq1, was evolved via an unusual frequenin gene duplication in *D*. melanogaster (Sánchez-Gracia et al. 2010). In *S. cerevisiae,* the *NCS-1* orthologue *FRQ1* plays a role in cell growth, and regulates a phosphatidylinositol-4-OH kinase (PIK-1) that is essential for cell survival (Hendricks et al. 1999). In *S. pombe, Ncs1p* has a role in the sexual development by regulation of spore formation and conjugation via Ca^2+^ dependent manner (Hamasaki-Katagiri et al. 2004). In *A. fumigatus*, the knockout mutantion of *ncs-1* homologue *ncsA* causes polarity defects, confers Ca^2+^ resistance, and increased sensitivity to EGTA, sodium dodecyl sulphate (SDS), ergosterol-depletion agents voriconazole and itraconazole, and the ergosterol intercalating agent amphotericin B; however, did not affect the virulence (Mota Júnior et al. 2008). NcsA also promotes expression of the *S. cerevisiae* Pmc1 homologs *pmcA*, a P-type Ca^2+^-ATPase, and *pmcB*, a Ca^2+^-translocating P-type ATPase in *A. fumigatus* (Mota Júnior et al. 2008; Soriani et al. 2008). In *M. grisea*, null-mutant of the *FRQ1*/*NCS-1*-like gene *Mg-NCS-1*, showed restricted growth at high Ca^2+^ concentrations or acidic conditions (Saitoh et al. 2003). The knockout mutant of *ncs-1* in *N. crassa* shows reduced growth and increased sensitivity to high concentrations of Ca^2+^ and UV irradiations (Tamuli et al. 2011; Deka et al. 2011). Besides, in response to the high concentrations of Ca^2+^, transcription of *ncs-1* is upregulated by the Crz-1 transcription factor in *N. crassa* (Gohain and Tamuli 2019). Furthermore, NCS-1 interacts with the Ca^2+^ permeable channel MID-1 in the plasma membrane, possibly to block Ca^2+^ influx, which might be critical for survival under the high concentration of Ca^2+^ (Gohain and Tamuli 2019).

## Calcineurin plays important roles in asexual and sexual developments, stress responses, and pathogenicity in fungi

Calcineurin was first identified as an inhibitor of CaM-dependent cyclic nucleotide phosphodiesterase in a column fraction (Wang and Desai 1976). Calcineurin is the only serine/threonine phosphatase that requires both Ca^2+^ and CaM for its activity (Klee et al. 1979, 1998). The heterodimeric enzyme calcineurin has two subunits, a catalytic subunit and a regulatory subunit (Klee and Krinks 1978). Calcineurin has a critical role in fungal development, stress responses, and virulence in pathogenic fungi (Rusnak and Mertz 2000; Juvvadi et al. 2014). The *S. cerevisiae MAT*a strains containing null-mutations in the calcineurin or protein phosphatase 2B (PP2B) subunits encoding genes *CNA1* and *CNA2* showed an increased sensitivity to growth arrest induced by the mating pheromone α-factor (Cyert et al. 1991). In *S. cerevisiae*, null mutations in both the calcineurin catalytic subunits (*cmp1cmp2* or *cna1cna2*), and the regulatory (*cnb1*) subunit, cause sensitivity to high concentrations of Mn^2+^ (MnCl_2_), because the mutants were not able to block the entry of Mn^2+^ into the cell (Farcasanu et al. 1995). In *N. crassa*, the RIP-generated *cnb-1* mutant showed a defect in hyphal growth and differentiation (Kothe and Free 1998). Inhibition of *cna-1* using antisense RNA and inhibitor of calcineurin FK506, results in a loss of steep Ca^2+^ gradient at the hyphal tip in *N. crassa* (Prokisch et al. 1997). Furthermore, the RIP-generated *Cna1* and *cnb-1* mutants displayed reduced thermotolerance, increased sensitivity to osmotic stress, and defect in the asexual and sexual developments in *N. crassa* (Kumar et al. 2019). In *M. oryzae*, application of antisense RNA against the catalytic subunit of calcineurin MCNA exhibited lessening in mycelial formation, conidiation, and appressorium formation, resulting in the reduction of the fungal pathogenicity (Choi et al. 2009a). In *C. neoformans*, the calcineurin catalytic subunit *CNA1* is required for growth at elevated temperature, survival in increased levels of CO_2_ and alkaline pH conditions, cation homoeostasis, and virulence (Odom et al. 1997). In the entomopathogenic fungus *Beauveria bassiana*, the *cnA1* and *cnA2* genes encode for two calcineurin catalytic subunit paralogues CnA1 and CnA2, and the *cnB* gene encodes the calcineurin regulatory subunit B (CnB) (Li et al. 2015). The *B. bassiana ∆cnA1, ∆cnA2*, and *∆cnB* deletion mutants showed reduced growth and conidiation, decreased virulence, sensitivity to stress-inducing chemicals and the fungicide carbendazim, and reduced survival in response to heat-shock, UV-B irradiation (Li et al. 2015). In addition, the *∆cnA1* and *∆cnA2* mutants showed altered cell wall composition, and the Δ*cnB* mutant showed sensitivity to osmotic stress induced by NaCl and KCl (Li et al. 2015). In *A. fumigatus*, calcineurin regulates hyphal growth, septation, and virulence (Lamoth et al. 2013; Juvvadi et al. 2014). In *S. pombe*, analysis of a null mutant of calcineurin-like gene *ppb1^+^* showed its involvement in cytokinesis, pole body positioning, cell shape and polarity, and mating (Yoshida et al. 1994).

In addition, calcineurin plays a critical role in fungal pathogenicity. *A. fumigatus* causes a common life-threatening disease called aspergillosis in humans. In the *Aspergillus* and *Candida* species, the antifungal paradoxical effect is a phenomenon, where reversal of growth inhibition occurs at higher concentrations of the antifungal drug echinocandins, usually caspofungin, which inhibit β-1,3-glucan synthase (*FKS1* in *C. albicans*, and FksA in *A. fumigatus*) causing damage of the fungal cell walls (Sanglard et al. 2003; Steinbach et al. 2015). The key players in the paradoxical effect are calcineurin catalytic subunit (CnaA) and the heat shock protein 90 (Hsp90) in *A. fumigatus* and *C. albicans* (Steinbach et al. 2015). Furthermore, the *A. fumigatus* calcineurin upregulates chitin biosynthesis, which causes increased chitin content in the cell wall, by transcriptional regulation of chitin synthase genes during paradoxical effect in response to high concentrations of caspofungin (Fortwendel et al. 2010). In *A. fumigatus*, caspofungin transiently increases [Ca^2+^]_c_ concentration, and activates CaM-calcineurin signalling, which causes paradoxical effect (Juvvadi et al. 2015). The *A. fumigatus* calcineurin catalytic A subunit (CnaA) contains a serine-proline-rich-region (SPRR) unique to the filamentous fungi, but absent in the human calcineurin α-catalytic subunit (Juvvadi et al. 2013). In the SPRR, phosphorylation of S406, S408, S410, and S413 residues are required for the function of calcineurin in hyphal growth and virulence of the *A. fumigatus* (Juvvadi et al. 2013). Identification of this critical SPRR region unique to filamentous fungi, but absent in human, is a significant step towards the development of new antifungal drugs for invasive aspergillosis (Juvvadi et al. 2013, 2014). Moreover, at paradoxical-growth concentrations of caspofugin (4 μg/ml), phosphorylation of S406, S410, and S413 residues in the SPRR of CnaA activates calcineurin, which may cause nuclear localisation of the transcription factor calcineurin responsive zinc finger 1 homologue CrzA for transcriptional activation to regulate paradoxical growth (Juvvadi et al. 2015). *CrzA* also has a role in chitin synthase expression in caspofungin paradoxical effect (CPE) in *A. fumigatus* (Ries et al. 2017). The pathways mediated by the CrzA and ZipD, which is a basic leucine zipper transcription factor and another target of calcineurin, genetically interact during Ca^2+^ stress (de Castro et al. 2019). Furthermore, in *A. fumigatus*, ZipD regulates cell wall composition and organisation, tolerance to osmotic stress, pathogenesis, and resistance to echinocandin antifungals, including caspofungin (de Castro et al. 2019). Therefore, targeting calcineurin CrzA and ZipD transcription factors may be potential drug targets against *A. fumigatus*. In addition, the role of CrzA has been investigated for differentiation and mycotoxin production in aflatoxin producing fungi *A. flavus* and *A. parasiticus* (Chang 2008; Lim et al. 2019). In *M. oryzae*, the calcineurin catalytic subunit A (*MCNA*) plays a role in mycelial growth, conidiation, formation of specialised infection structure called appressorium, and pathogenicity (Choi et al. 2009a). Moreover, in *M. oryzae*, the knock-down of catalytic subunit A-like gene moderately reduced appressorium formation, but the knock-down of regulatory subunit B-like gene causes a complete loss of pathogenicity against the host plants, suggesting that the catalytic and regulatory subunits play a distinct role in pathogenicity (Nguyen et al. 2008). In *C. neoformans*, Δ*cna1* deletion mutants were unable to survive at a body temperature of 37°C, alkaline pH, and CO_2_ at high levels (Odom et al. 1997). The *C. neoformans* virulence was studied using an immunosuppressed rabbit, an animal model of cryptococcal meningitis (Odom et al. 1997). The virulence was examined by removal of cerebral blood spinal fluid (CSF) and counting of the colony-forming units (CFU), which revealed a low number CFU in the Δ*cna1* mutant of *C. neoformans*, indicating that calcineurin is required for growth in the mammalian host (Odom et al. 1997). Similarly, disruption of *cnb1* in *C. neoformans* results in temperature-sensitivity and reduced virulence in the murine model of cryptococcosis (Fox et al. 2001). Calcineurin in *C. neoformans* was identified as a novel antifungal drug target (Cruz et al. 2000). *C. neoformans* is sensitive to FK506 and CsA at physiological temperature (Cruz et al. 2000). A mutational analysis using a novel 6 bp duplication in the calcineurin B gene (*CNB1*) inhibits the immunophilins FKBP12 – FK506 binding to Cnb1, which revealed the mechanism of drug action in *C. neoformans* (Fox et al. 2001). Calcineurin A is also vital for providing tolerance to antifungal agents along with some metabolic inhibitors in *C. albicans* (Cruz et al. 2002). In *C. albicans*, disruption of *cna-1* results in the loss of viability in the presence of antifungal agents fluconazole, amorolfine, itraconazole, terbinafine, and voriconazole (Sanglard et al. 2003). In addition to calcineurin, Hsp90 has a role in providing resistance to echinocandins in *C. albicans* (Singh et al. 2009). Calcineurin inhibitors, cyclosporine and fluconazole have a synergistic effect in the prevention of biofilm formation and increase susceptibility to fluconazole in *C. albicans* (Jia et al. 2016).

## Calcineurin responsive zinc finger 1 (Crz1) plays a critical role in regulating cellular functions, tolerance to stress conditions, and virulence

There are different target proteins of calcineurin found across different organisms (Li et al. 2011). The best-known target is a transcription factor called calcineurin-responsive zinc finger 1 (Crz1) in lower eukaryotes and the nuclear factor of activated T cells (NFAT) in mammals (Thewes 2014). In *S. cerevisiae*, the calcineurin target Crz1p, also known as Tcn1p, drives the expressions of PMC1, PMR1, PMR2A, and FKS2 genes, which play a vital role in the tolerance to high Ca^2+^, Mn^2+^, Na^+^, and cell wall damage, respectively (Matheos et al. 1997; Stathopoulos and Cyert 1997). In *S. pombe*, the null mutant of *prz1*, the *crz1* homologue, did not show any defects in morphology and sexual development; however, the Δ*prz1* mutant was hypersensitive to Ca^2+^ (Hirayama et al. 2003). In *M. grisea*, the CRZ1 homologue MgCRZ1 partially complemented the *S. cerevisiae Δcrz1* mutant and suppressed the Li^+^ sensitivity (Zhang et al. 2009). The MgCRZ1 is required for growth, development, Ca^2+^ stress tolerance, and full virulence (Zhang et al. 2009). In *C. neoformans, crz1* drives the expression of the chitin synthase gene *chs6*, and *crz1* deletion mutant showed a defect in cell wall synthesis (Lev et al. 2012). In *C. albicans*, the *CRZ1* deletion mutant was more sensitive to Ca^2+^, Li^+^, and Mn^2+^ cations, hypersensitive to anionic detergents SDS and antifungal azoles, including fluconazole and miconazole (Santos and de Larrinoa 2005). In *Aspergillus parasiticus, crzA* is required for vegetative growth, asexual development, and aflatoxin production under the Ca^2+^ stress condition (Chang 2008). In *A. fumigatus*, knock out of *crzA* results in a defect in germination and polarised hyphal growth, and reduced sporulation (Cramer et al. 2008). The *A. fumigatus* Δ*crzA* mutants also displayed sensitivity to heat shock conditions (Soriani et al. 2008). Studies in *A. flavus*, which produces aflatoxin, the deletion of *crzA* renders the strain more vulnerable to cell wall stress and osmotic pressure (Lim et al. 2019). In the *A. flavus* Δ*crzA* mutants, conidiophore production was reduced as the head of the conidiophore become short, along with a decrease in the number of conidia (Lim et al. 2019).

Crz 1 also plays a critical role in fungal virulence. In *M. grisea*, when infected to onion surface or rice leaves, Δ*mocrz1* showed a significant reduction in the appressorial penetration rate (Choi et al. 2009b). In *M. grisea*, lipid droplets are transported from conidia to nascent appressorium, which fully melanised within 12–24 h (Zhang et al. 2009). However, in the *M. grisea* Δ*crz1* mutant, the lipid droplets were undegraded even after 48 h (Zhang et al. 2009). Thus, targeting calcineurin-CRZ1 signalling cascade results in a lack of functional appressorium that failed to penetrate the host cuticle, causing loss of full virulence in *M. grisea* (Zhang et al. 2009). In *B. bassiana*, Δ*crz1* mutant showed defects in growth and conidiation, suppressed growth in the presence of osmotic salts, sensitivity to stress-inducing chemicals and carbendazim and osmotic salts, decreased thermotolerance, reduced resistance to UV-B irradiation, changed cell wall composition, and longer virulence period (Li et al. 2015).

## Conclusions

Ca^2+^ signalling regulates multiple cell functions ranging from growth, development, fertility, stress tolerance, and virulence in fungi. Phospholipases are enzymes that act on membrane phospholipids and classified into four types. In response to specific signals, PLC produces IP_3_ and DAG, which mediate Ca^2+^ release and activation of PKC, respectively. There are also six major types of Ca^2+^ transporters that are required for Ca^2+^ homoeostasis and signalling. The increase in [Ca^2+^]_c_ activates various Ca^2+^ sensors such as CaM and NCS-1 that interact with specific downstream proteins as a response to a signal. Calcineurin, which consists of catalytic A and regulatory B subunits, is one of the major downstream Ca^2+^ signalling proteins required for multiple cell functions, including growth, stress tolerance, and virulence in fungi. Calcineurin is also identified as a target for antifungal drugs. Calcineurin activates the Crz 1 transcription factor to activate expressions of target genes required for growth, developments, tolerance to stress conditions, and pathogenicity. Further research on the Ca^2+^ signalling machinery will unravel its complex molecular network under different cellular conditions.

## References

[cit0001] Andaluz E, Coque JR, Cueva R, Larriba G. 2001. Sequencing of a 4.3 kbp region of chromosome 2 of *Candida albicans* reveals the presence of homologues of *SHE9* from Saccharomyces cerevisiae and of bacterial phosphatidylinositol‐phospholipase C. Yeast. 18(8):711–721. doi:10.1002/yea.716. 11378898

[cit0002] Bahn Y-S, Jung K-W. 2013. Stress signaling pathways for the pathogenicity of *Cryptococcus*. Eukaryot Cell. 12(12):1564–1577. doi:10.1128/EC.00218-13. 24078305 PMC3889573

[cit0003] Barman A, Gohain D, Bora U, Tamuli R. 2018. Phospholipases play multiple cellular roles including growth, stress tolerance, sexual development, and virulence in fungi. Microbiol Res. 209:55–69. doi:10.1016/j.micres.2017.12.012 29580622

[cit0004] Barman A, Tamuli R. 2015. Multiple cellular roles of *Neurospora crassa plc-1, splA2*, and *cpe-1* in regulation of cytosolic free calcium, carotenoid accumulation, stress responses, and acquisition of thermotolerance. J Microbiol. 53(4):226–235. doi:10.1007/s12275-015-4465-1. 25636422

[cit0005] Barman A, Tamuli R. 2017. The pleiotropic vegetative and sexual development phenotypes of *Neurospora crassa* arise from double mutants of the calcium signaling genes *plc-1, splA2*, and *cpe-1*. Curr Genet. 63(5):861–875. doi:10.1007/s00294-017-0682-y. 28265741

[cit0006] Bates S, Maccallum DM, Bertram G, Munro CA, Hughes HB, Buurman ET, Brown AJP, Odds FC, Gow NAR. 2005. *Candida albicans* Pmr1p, a secretory pathway P-type Ca^2+^/Mn^2+^ - ATPase, is required for glycosylation and virulence. J Biol Chem. 280(24):23408–23415. doi:10.1074/jbc.M502162200. 15843378

[cit0007] Bennett DE, McCreary CE, Coleman DC. 1998. Genetic characterization of a phospholipase C gene from *Candida albicans*: presence of homologous sequences in *Candida* species other than *Candida albicans*. Microbiol. 144(1):55–72. doi:10.1099/00221287-144-1-55. 9467900

[cit0008] Berridge MJ. 1993. Inositol trisphosphate and calcium signalling. Nature. 361(6410):315. doi:10.1038/361315a0. 8381210

[cit0009] Berridge MJ. 1998. Neuronal calcium signaling. Neuron. 21(1):13–26. doi:10.1016/S0896-6273(00)80510-3. 9697848

[cit0010] Berridge MJ, Bootman MD, Lipp P. 1998. Calcium-a life and death signal. Nature. 395(6703):645–648. doi:10.1038/27094. 9790183

[cit0011] Berridge MJ, Bootman MD, Roderick HL. 2003. Calcium: calcium signalling: dynamics, homeostasis and remodelling. Nat Rev Mol Cell Biol. 4(7):517. doi:10.1038/nrm1155. 12838335

[cit0012] Birch M, Robson G, Law D, Denning DW. 1996. Evidence of multiple extracellular phospholipase activities of *Aspergillus fumigatus*. Infect Immun. 64(3):751–755. doi:10.1128/IAI.64.3.751-755.1996. 8641777 PMC173833

[cit0013] Borkovich KA, Alex LA, Yarden O, Freitag M, Turner GE, Read ND, Seiler S, Bell-Pedersen D, Paietta J, Plesofsky N, et al. 2004. Lessons from the genome sequence of *Neurospora crassa*: tracing the path from genomic blueprint to multicellular organism. Microbiol Mol Biol Rev. 68(1):1–108. 15007097 10.1128/MMBR.68.1.1-108.2004 PMC362109

[cit0014] Bowman BJ, Abreu S, Margolles-clark E, Draskovic M, Bowman EJ. 2011. Role of four calcium transport proteins, encoded by *nca-1*, *nca-2*, *nca-3*, and *cax*, in maintaining intracellular calcium levels in *Neurospora crassa*. Eukaryot Cell. 10(5):654–661. doi:10.1128/EC.00239-10. 21335528 PMC3127652

[cit0015] Brini M, Calì T, Ottolini D, Carafoli E. 2013a. Intracellular calcium homeostasis and signaling. In: Banci L, editor. Met Ions Life Sci. Vol. 12. Dordrecht: Springer; p. 119–168. 10.1007/978-94-007-5561-1_5 23595672

[cit0016] Brini M, Calì T, Ottolini D, Carafoli E. 2013b. The plasma membrane calcium pump in health and disease. Febs J. 280(21):5385–5397. doi:10.1111/febs.12193. 23413890

[cit0017] Burgoyne RD. 2007. Neuronal calcium sensor proteins: generating diversity in neuronal Ca^2+^ signalling. Nat Rev Neurosci. 8(3):182. doi:10.1038/nrn2093. 17311005 PMC1887812

[cit0018] Carafoli E. 2002. Calcium signaling: a tale for all seasons. Proc Natl Acad Sci. 99(3):1115–1122. doi:10.1073/pnas.032427999. 11830654 PMC122154

[cit0019] Case RM, Eisner D, Gurney A, Jones O, Muallem S, Verkhratsky A. 2007. Evolution of calcium homeostasis: from birth of the first cell to an omnipresent signalling system. Cell Calcium. 42(4–5):345–350. doi:10.1016/j.ceca.2007.05.001. 17574670

[cit0020] Chae SW, Kim J-M, Yun YP, Lee WK, Kim J-S, Kim Y-H, Lee K-S, Ko YJ, Lee K-H, Rha HK. 2007. Identification and analysis of the promoter region of the human PLC-*δ*4 gene. Mol Biol Rep. 34(2):69–77. doi:10.1007/s11033-006-9014-x. 17394098

[cit0021] Chang P-K. 2008. *Aspergillus parasiticus crzA*, which encodes calcineurin response zinc-finger protein, is required for aflatoxin production under calcium stress. Int J Mol Sci. 9(10):2027–2043. doi:10.3390/ijms9102027. 19325734 PMC2635607

[cit0022] Chin D, Means AR. 2000. Calmodulin: a prototypical calcium sensor. Trends Cell Biol. 10(8):322–328. doi:10.1016/S0962-8924(00)01800-6. 10884684

[cit0023] Choi J, Kim Y, Kim S, Park J, Lee Y-H. 2009b. *MoCRZ1*, a gene encoding a calcineurin-responsive transcription factor, regulates fungal growth and pathogenicity of *Magnaporthe oryzae*. Fungal Genet Biol. 46(3):243–254. doi:10.1016/j.fgb.2008.11.010. 19111943

[cit0024] Choi J-H, Kim Y-S, Lee Y-H. 2009a. Functional analysis of *MCNA*, a gene encoding a catalytic subunit of calcineurin, in the rice blast fungus *Magnaporthe oryzae*. J Microbiol Biotechnol. 19(1):11–16. 19190403

[cit0025] Clapham DE. 2007. Calcium signaling. Cell. 131(6):1047–1058. doi:10.1016/j.cell.2007.11.028. 18083096

[cit0026] Cornelius G, Nakashima H. 1987. Vacuoles play a decisive role in calcium homeostasis *Neurospora crassa* . Microbio. 133(8):2341–2347.

[cit0027] Cortés JCG, Katoh-Fukui R, Moto K, Ribas JC, Ishiguro J. 2004. *Schizosaccharomyces pombe* Pmr1p is essential for cell wall integrity and is required for polarized cell growth and cytokinesis. Eukaryot Cell. 3(5):1124–1135. doi:10.1128/EC.3.5.1124-1135.2004. 15470240 PMC522595

[cit0028] Cramer RA, Perfect BZ, Pinchai N, Park S, Perlin DS, Asfaw YG, Heitman J, Perfect JR, Steinbach WJ. 2008. Calcineurin target CrzA regulates conidial germination, hyphal growth, and pathogenesis of *Aspergillus fumigatus*. Eukaryot Cell. 7(7):1085–1097. doi:10.1128/EC.00086-08. 18456861 PMC2446674

[cit0029] Cruz MC, Del Poeta M, Wang P, Wenger R, Zenke G, Quesniaux VFJ, Movva NR, Perfect JR, Cardenas ME, Heitman J. 2000. Immunosuppressive and nonimmunosuppressive cyclosporine analogs are toxic to the opportunistic fungal pathogen *Cryptococcus neoformans* via cyclophilin-dependent inhibition of calcineurin. Antimicrob Agents Chemother. 44(1):143–149. doi:10.1128/AAC.44.1.143-149.2000. 10602736 PMC89641

[cit0030] Cruz MC, Goldstein AL, Blankenship JR, Del Poeta M, Davis D, Cardenas ME, Perfect JR, McCusker JH, Heitman J. 2002. Calcineurin is essential for survival during membrane stress in *Candida albicans*. Embo J. 21(4):546–559. doi:10.1093/emboj/21.4.546. 11847103 PMC125859

[cit0031] Cunningham KW, Fink GR. 1994. Calcineurin-dependent growth control in *Saccharomyces cerevisiae* mutants lacking *PMC1*, a homolog of plasma membrane Ca^2+^ ATPases. J Cell Biol. 124(3):351–363. doi:10.1083/jcb.124.3.351. 7507493 PMC2119937

[cit0032] Cyert MS, Kunisawa R, Kaim D, Thorner J. 1991. Yeast has homologs (*CNA1* and *CNA2* gene products) of mammalian calcineurin, a calmodulin-regulated phosphoprotein phosphatase. Proc Natl Acad Sci. 88(16):7376–7380. doi:10.1073/pnas.88.16.7376. 1651503 PMC52298

[cit0033] Davis TN, Urdea MS, Masiarz FR, Thorner J. 1986. Isolation of the yeast calmodulin gene: calmodulin is an essential protein. Cell. 47(3):423–431. doi:10.1016/0092-8674(86)90599-4. 3533275

[cit0034] Davis TR, Zucchi PC, Kumamoto CA. 2013. Calmodulin binding to Dfi1p promotes invasiveness of *Candida albicans*. PLoS One. 8(10):e76239. doi:10.1371/journal.pone.0076239. 24155896 PMC3796530

[cit0035] de Castro PA, Chiaratto J, Winkelströter LK, Bom VLP, Ramalho LNZ, Goldman MHS, Brown NA, Goldman GH. 2014. The involvement of the Mid1/Cch1/Yvc1 calcium channels in *Aspergillus fumigatus* virulence. PLoS One. 9(8):e103957. doi:10.1371/journal.pone.0103957. 25083783 PMC4118995

[cit0036] de Castro PA, Colabardini AC, Manfiolli AO, Chiaratto J, Silva LP, Mattos EC, Palmisano G, Almeida F, Persinoti GF, Ries LNA. 2019. *Aspergillus fumigatus* calcium-responsive transcription factors regulate cell wall architecture promoting stress tolerance, virulence and caspofungin resistance. PLoS Genet. 15(12):12. doi:10.1371/journal.pgen.1008551. PMC6948819 31887136

[cit0037] Dean RA, Talbot NJ, Ebbole DJ, Farman ML, Mitchell TK, Orbach MJ, Thon M, Kulkarni R, Xu J-R, Pan H. 2005. The genome sequence of the rice blast fungus *Magnaporthe grisea*. Nature. 434(7036):980. doi:10.1038/nature03449. 15846337

[cit0038] Deka R, Kumar R, Tamuli R. 2011. *Neurospora crassa* homologue of neuronal calcium sensor-1 has a role in growth, calcium stress tolerance, and ultraviolet survival. Genetica. 139(7):885–894. doi:10.1007/s10709-011-9592-y. 21728141

[cit0039] Deka R, Tamuli R. 2013. *Neurospora crassa ncs-1, mid-1* and *nca-2* double-mutant phenotypes suggest diverse interaction among three Ca^2+^-regulating gene products. J Genet. 92(3):559–563. doi:10.1007/s12041-013-0270-y. 24371176

[cit0040] Dinamarco TM, Freitas FZ, Almeida RS, Brown NA, Dos Reis TF, Ramalho LNZ, Savoldi M, Goldman MHS, Bertolini MC, Goldman GH. 2012. Functional characterization of an *Aspergillus fumigatus* calcium transporter (PmcA) that is essential for fungal infection. PLoS One. 7(5):e37591. doi:10.1371/journal.pone.0037591. 22649543 PMC3359301

[cit0041] Engel SR, Dietrich FS, Fisk DG, Binkley G, Balakrishnan R, Costanzo MC, Dwight SS, Hitz BC, Karra K, Nash RS. 2014. The reference genome sequence of *Saccharomyces cerevisiae*: then and now. G3 genes, genomes. Genet. 4(3):389–398. 10.1534/g3.113.008995 PMC3962479 24374639

[cit0042] Fan Y, Ortiz-Urquiza A, Kudia RA, Keyhani NO. 2012. A fungal homologue of neuronal calcium sensor-1, *Bbcsa1*, regulates extracellular acidification and contributes to virulence in the entomopathogenic fungus *Beauveria bassiana*. Microbiol. 158(7):1843–1851. doi:10.1099/mic.0.058867-0. 22504440

[cit0043] Fankhauser H, Schweingruber AM, Edenharter E, Schweingruber ME. 1995. Growth of a mutant defective in a putative phosphoinositide-specific phospholipase C of *Schizosaccharomyces pombe* is restored by low concentrations of phosphate and inositol. Curr Genet. 28(2):199–203. doi:10.1007/BF00315789. 8590474

[cit0044] Farcasanu IC, Hirata D, Tsuchiya E, Nishiyama F, Miyakawa T. 1995. Protein phosphatase 2B of *Saccharomyces cerevisiae* is required for tolerance to manganese, in blocking the entry of ions into the cells. Eur J Biochem. 232(3):712–717. doi:10.1111/j.1432-1033.1995.tb20865.x. 7588708

[cit0045] Flick JS, Thorner J. 1993. Genetic and biochemical characterization of a phosphatidylinositol-specific phospholipase C in *Saccharomyces cerevisiae*. Mol Cell Biol. 13(9):5861–5876. doi:10.1128/MCB.13.9.5861. 8395015 PMC360334

[cit0046] Fortwendel JR, Juvvadi PR, Perfect BZ, Rogg LE, Perfect JR, Steinbach WJ. 2010. Transcriptional regulation of chitin synthases by calcineurin controls paradoxical growth of *Aspergillus fumigatus* in response to caspofungin. Antimicrob Agents Chemother. 54(4):1555–1563. doi:10.1128/AAC.00854-09. 20124000 PMC2849361

[cit0047] Fox DS, Cruz MC, Sia RAL, Ke H, Cox GM, Cardenas ME, Heitman J. 2001. Calcineurin regulatory subunit is essential for virulence and mediates interactions with FKBP12–FK506 in *Cryptococcus neoformans*. Mol Microbiol. 39(4):835–849. doi:10.1046/j.1365-2958.2001.02295.x. 11251806

[cit0048] Galagan JE, Calvo SE, Borkovich KA, Selker EU, Read ND, Jaffe D, FitzHugh W, Ma L-J, Smirnov S, Purcell S, et al. 2003. The genome sequence of the filamentous fungus *Neurospora crassa*. Nature. 422(6934):859–868. doi:10.1038/nature01554 12712197

[cit0049] Galimov E. 2009. Concept of sustained ordering and an ATP-related mechanism of life’s origin. Int J Mol Sci. 10(5):2019–2030. doi:10.3390/ijms10052019. 19564936 PMC2695264

[cit0050] Gavric O, Dos Santos DB, Griffiths A. 2007. Mutation and divergence of the phospholipase C gene in *Neurospora crassa*. Fungal Genet Biol. 44(4):242–249. doi:10.1016/j.fgb.2006.09.010. 17157541

[cit0051] Gohain D, Tamuli R. 2019. Calcineurin responsive zinc‐finger‐1 binds to a unique promoter sequence to upregulate neuronal calcium sensor‐1, whose interaction with MID‐1 increases tolerance to calcium stress in *Neurospora crassa*. Mol Microbiol. 111(6):1510–1528. doi:10.1111/mmi.14234. 30825330

[cit0052] Halling DB, Liebeskind BJ, Hall AW, Aldrich RW. 2016. Conserved properties of individual Ca^2+^-binding sites in calmodulin. Proc Natl Acad Sci. 113(9):E1216–E1225. doi:10.1073/pnas.1600385113. 26884197 PMC4780646

[cit0053] Hamasaki-Katagiri N, Molchanova T, Takeda K, Ames JB. 2004. Fission yeast homolog of neuronal calcium sensor-1 (Ncs1p) regulates sporulation and confers calcium tolerance. J Biol Chem. 279(13):12744–12754. doi:10.1074/jbc.M311895200. 14722091

[cit0054] Hamilton SL. 2005. Ryanodine receptors. Cell Calcium. 38(3–4):253–260. doi:10.1016/j.ceca.2005.06.037. 16115682

[cit0055] Hendricks KB, Wang BQ, Schnieders EA, Thorner J. 1999. Yeast homologue of neuronal frequenin is a regulator of phosphatidylinositol-4-OH kinase. Nat Cell Biol. 1(4):234–241. doi:10.1038/12058. 10559922

[cit0056] Hirayama S, Sugiura R, Lu Y, Maeda T, Kawagishi K, Yokoyama M, Tohda H, Giga-Hama Y, Shuntoh H, Kuno T. 2003. Zinc finger protein prz1 regulates Ca^2+^ but not Cl^−^ homeostasis in fission yeast. Identification of distinct branches of calcineurin signaling pathway in fission yeast. J Biol Chem. 278(20):18078–18084. doi:10.1074/jbc.M212900200. 12637524

[cit0057] Hong Y, Zhao J, Guo L, Kim S-C, Deng X, Wang G, Zhang G, Li M, Wang X. 2016. Plant phospholipases D and C and their diverse functions in stress responses. Prog Lipid Res. 62:55–74. doi:10.1016/j.plipres.2016.01.002. 26783886

[cit0058] Itadani A, Nakamura T, Hirata A, Shimoda C. 2010. *Schizosaccharomyces pombe* calmodulin, Cam1, plays a crucial role in sporulation by recruiting and stabilizing the spindle pole body components responsible for assembly of the forespore membrane. Eukaryot Cell. 9(12):1925–1935. doi:10.1128/EC.00022-10. 20833892 PMC3008279

[cit0059] Jaiswal JK. 2001. Calcium—how and why? J Biosci. 26(3):357–363. doi:10.1007/BF02703745. 11568481

[cit0060] Jia W, Zhang H, Li C, Li G, Liu X, Wei J. 2016. The calcineruin inhibitor cyclosporine a synergistically enhances the susceptibility of *Candida albicans* biofilms to fluconazole by multiple mechanisms. BMC Microbiol. 16(1):113. doi:10.1186/s12866-016-0728-1. 27316338 PMC4912705

[cit0061] Jones T, Federspiel NA, Chibana H, Dungan J, Kalman S, Magee BB, Newport G, Thorstenson YR, Agabian N, Magee PT. 2004. The diploid genome sequence of *Candida albicans*. Proc Natl Acad Sci. 101(19):7329–7334. doi:10.1073/pnas.0401648101. 15123810 PMC409918

[cit0062] Juvvadi PR, Gehrke C, Fortwendel JR, Lamoth F, Soderblom EJ, Cook EC, Hast MA, Asfaw YG, Moseley MA, Creamer TP. 2013. Phosphorylation of calcineurin at a novel serine-proline rich region orchestrates hyphal growth and virulence in *Aspergillus fumigatus*. PLoS Pathog. 9(8):e1003564. doi:10.1371/journal.ppat.1003564. 23990785 PMC3749960

[cit0063] Juvvadi PR, Lamoth F, Steinbach WJ. 2014. Calcineurin as a multifunctional regulator: unraveling novel functions in fungal stress responses, hyphal growth, drug resistance, and pathogenesis. Fungal Biol Rev. 28(2–3):56–69. doi:10.1016/j.fbr.2014.02.004. 25383089 PMC4219591

[cit0064] Juvvadi PR, Muñoz A, Lamoth F, Soderblom EJ, Moseley MA, Read ND, Steinbach WJ. 2015. Calcium-mediated induction of paradoxical growth following caspofungin treatment is associated with calcineurin activation and phosphorylation in *Aspergillus fumigatus*. Antimicrob Agents Chemother. 59(8):4946–4955. doi:10.1128/AAC.00263-15. 26055379 PMC4505252

[cit0065] Kellermayer R, Aiello DP, Miseta A, Bedwell DM. 2003. Extracellular Ca^2+^ sensing contributes to excess Ca^2+^ accumulation and vacuolar fragmentation in a *pmr1∆* mutant of *S. cerevisiae*. J Cell Sci. 15;116(8):1637–1646. doi:10.1242/jcs.00372. 12640047

[cit0066] Klee CB, Crouch TH, Krinks MH. 1979. Calcineurin: A calcium- and calmodulin-binding protein of the nervous system. Proc Natl Acad Sci U S A. 76(12):6270–6273. doi:10.1073/pnas.76.12.6270. 293720 PMC411845

[cit0067] Klee CB, Krinks MH. 1978. Purification of cyclic 3ʹ, 5ʹ-nucleotide phosphodiesterase inhibitory protein by affinity chromatography on activator protein coupled to sepharose. Biochemistry. 17(1):120–126. doi:10.1021/bi00594a017. 201280

[cit0068] Klee CB, Ren H, Wang X. 1998. Regulation of the calmodulin-stimulated protein phosphatase, calcineurin. J Biol Chem. 273(22):13367–13370. doi:10.1074/jbc.273.22.13367. 9593662

[cit0069] Kmetzsch L, Staats CC, Rodrigues ML, Schrank A, Vainstein MH. 2011. Calcium signaling components in the human pathogen *Cryptococcus neoformans: Cryptococcus neoformans*. Commun Integr Biol. 4(2):186–187. doi:10.4161/cib.4.2.14271. 21655435 PMC3104574

[cit0070] Knechtle P, Goyard S, Brachat S, Ibrahim-Granet O, D’Enfert C. 2005. Phosphatidylinositol-dependent phospholipases C Plc2 and Plc3 of *Candida albicans* are dispensable for morphogenesis and host–pathogen interaction. Res Microbiol. 156(7):822–829. doi:10.1016/j.resmic.2005.04.007. 16040234

[cit0071] Köhler GA, Brenot A, Haas-Stapleton E, Agabian N, Deva R, Nigam S. 2006. Phospholipase A2 and phospholipase B activities in fungi. Biochim Biophys Acta (BBA)-Mol Cell Biol Lipids. 1761(11):1391–1399. 10.1016/j.bbalip.2006.09.011 PMC2077850 17081801

[cit0072] Kothe GO, Free SJ. 1998. Calcineurin subunit B is required for normal vegetative growth in *Neurospora crassa*. Fungal Genet Biol. 23(3):248–258. doi:10.1006/fgbi.1998.1037. 9680955

[cit0073] Kraus PR, Nichols CB, Heitman J. 2005. Calcium- and calcineurin-independent roles for calmodulin in Cryptococcus neoformans morphogenesis and high-temperature growth. Eukaryot Cell. 4(6):1079–1087. doi:10.1128/EC.4.6.1079-1087.2005 PMC1151996 15947200

[cit0074] Kumar A, Roy A, Deshmukh MV, Tamuli R. 2019. Dominant mutants of the calcineurin catalytic subunit (CNA-1) showed developmental defects, increased sensitivity to stress conditions, and CNA-1 interacts with CaM and CRZ-1 in *Neurospora crassa*. Arch Microbiol. 202(4):921–934. doi:10.1007/s00203-019-01768-z. 31807806

[cit0075] Kumar R, Tamuli R. 2014. Calcium/calmodulin-dependent kinases are involved in growth, thermotolerance, oxidative stress survival, and fertility in *Neurospora crassa*. Arch Microbiol. 196(4):295–305. doi:10.1007/s00203-014-0966-2. 24570326

[cit0076] Kunze D, Melzer I, Bennett D, Sanglard D, MacCallum D, Nörskau J, Coleman DC, Odds FC, Schäfer W, Hube B. 2005. Functional analysis of the phospholipase C gene *CaPLC1* and two unusual phospholipase C genes, *CaPLC2* and *CaPLC3*, of *Candida albicans*. Microbiol. 151(10):3381–3394. doi:10.1099/mic.0.28353-0. 16207920

[cit0077] Lamoth F, Juvvadi PR, Gehrke C, Steinbach WJ. 2013. vitro activity of calcineurin and heat shock protein 90 inhibitors against *Aspergillus fumigatus* azole-and echinocandin-resistant strains. Antimicrob Agents Chemother. 57(2):1035–1039. doi:10.1128/AAC.01857-12. 23165466 PMC3553695

[cit0078] Lange M, Peiter E. 2020. Calcium transport proteins in fungi: the phylogenetic diversity of their relevance for growth, virulence, and stress resistance. Front Microbiol. 10:3100. doi:10.3389/fmicb.2019.03100. 32047484 PMC6997533

[cit0079] Laxmi V, Tamuli R. 2015. The *Neurospora crassa cmd, trm-9*, and *nca-2* genes play a role in growth, development, and survival in stress conditions. Gen Appl Biol. 6(7):1–8. doi:10.5376/gab.2015.06.0007.

[cit0080] Laxmi V, Tamuli R. 2017. The calmodulin gene in *Neurospora crassa* is required for normal vegetative growth, ultraviolet survival, and sexual development. Arch Microbiol. 199(4):531–542. doi:10.1007/s00203-016-1319-0. 27888323

[cit0081] Lee K-T, So Y-S, Yang D-H, Jung K-W, Choi J, Lee D-G, Kwon H, Jang J, Wang LL, Cha S. 2016. Systematic functional analysis of kinases in the fungal pathogen *Cryptococcus neoformans*. Nat Commun. 7(1):12766. doi:10.1038/ncomms12766. 27677328 PMC5052723

[cit0082] Lee SC, Lee YH. 1998. Calcium/calmodulin-dependent signaling for appressorium formation in the plant pathogenic fungus *Magnaporthe grisea*. Mol Cells. 8(6):698–704. 9895122

[cit0083] Lev S, Desmarini D, Chayakulkeeree M, Sorrell TC, Djordjevic JT. 2012. The Crz1/Sp1 transcription factor of *Cryptococcus neoformans* is activated by calcineurin and regulates cell wall integrity. PLoS One. 7(12):e51403. doi:10.1371/journal.pone.0051403. 23251520 PMC3520850

[cit0084] Lev S, Desmarini D, Li C, Chayakulkeeree M, Traven A, Sorrell TC, Djordjevic JT. 2013. Phospholipase C of *Cryptococcus neoformans* regulates homeostasis and virulence by providing inositol trisphosphate as a substrate for Arg1 kinase. Infect Immun. 81(4):1245–1255. doi:10.1128/IAI.01421-12. 23381992 PMC3639591

[cit0085] Lew RR, Giblon RE, Lorenti MSH. 2015. The phenotype of a phospholipase C (*plc-1*) mutant in a filamentous fungus, *Neurospora crassa*. Fungal Genet Biol. 82:158–167. doi:10.1016/j.fgb.2015.07.007. 26212074

[cit0086] Lewit-Bentley A, Réty S. 2000. EF-hand calcium-binding proteins. Curr Opin Struct Biol. 10(6):637–643. doi:10.1016/S0959-440X(00)00142-1. 11114499

[cit0087] Li F, Wang Z-L, Zhang L-B, Ying S-H, Feng M-G. 2015. The role of three calcineurin subunits and a related transcription factor (Crz1) in conidiation, multistress tolerance and virulence in *Beauveria bassiana*. Appl Microbiol Biotechnol. 99(2):827–840. doi:10.1007/s00253-014-6124-6. 25324131

[cit0088] Li H, Rao A, Hogan PG. 2011. Interaction of calcineurin with substrates and targeting proteins. Trends Cell Biol. 21(2):91–103. doi:10.1016/j.tcb.2010.09.011. 21115349 PMC3244350

[cit0089] Li Y, Zhang Y, Lu L. 2019. Calcium signaling pathway is involved in non-CYP51 azole resistance in *Aspergillus fumigatus*. Med Mycol. 57(Supplement_2):S233–S238. doi:10.1093/mmy/myy075. 30816964

[cit0090] Lim S-Y, Son Y-E, Lee D-H, Eom T-J, Kim M-J, Park H-S. 2019. Function of crzA in fungal development and aflatoxin production in *Aspergillus flavus*. Toxins (Basel). 11(10):567. doi:10.3390/toxins11100567. PMC6832762 31569747

[cit0091] Liu Z-M, Kolattukudy PE. 1999. Early expression of the calmodulin gene, which precedes appressorium formation in *Magnaporthe grisea*, is inhibited by self-inhibitors and requires surface attachment. J Bacteriol. 181(11):3571–3577. doi:10.1128/JB.181.11.3571-3577.1999. 10348871 PMC93826

[cit0092] Luna-Tapia A, DeJarnette C, Sansevere E, Reitler P, Butts A, Hevener KE, Palmer GE. 2019. The vacuolar Ca^2+^ ATPase Pump *Pmc1p* Is required for *Candida albicans* pathogenesis. mSphere. 4(1):e00715–18. doi:10.1128/mSphere.00715-18. PMC6365616 30728284

[cit0093] Ma L-J, Van Der Does HC, Borkovich KA, Coleman JJ, Daboussi M-J, Di Pietro A, Dufresne M, Freitag M, Grabherr M, Henrissat B. 2010. Comparative genomics reveals mobile pathogenicity chromosomes in fusarium. Nature. 464(7287):367. doi:10.1038/nature08850. 20237561 PMC3048781

[cit0094] Martin DC, Kim H, Mackin NA, Maldonado-Báez L, Evangelista CC, Beaudry VG, Dudgeon DD, Naiman DQ, Erdman SE, Cunningham KW. 2011. New regulators of a high affinity Ca^2+^ influx system revealed through a genome-wide screen in yeast. J Biol Chem. 286(12):10744–10754. doi:10.1074/jbc.M110.177451. 21252230 PMC3060525

[cit0095] Matheos DP, Kingsbury TJ, Ahsan US, Cunningham KW. 1997. Tcn1p/Crz1p, a calcineurin-dependent transcription factor that differentially regulates gene expression in *Saccharomyces cerevisiae*. Genes Dev. 11(24):3445–3458. doi:10.1101/gad.11.24.3445. 9407036 PMC316804

[cit0096] Mikoshiba K, Hattori M. 2000. IP3 receptor-operated calcium entry. Sci STKE. 2000(51):–pe1. doi:10.1126/stke.2000.51.pe1. 11752610

[cit0097] Moser MJ, Flory MR, Davis TN. 1997. Calmodulin localizes to the spindle pole body of *Schizosaccharomyces pombe* and performs an essential function in chromosome segregation. J Cell Sci. 110(15):1805–1812. 9264467 10.1242/jcs.110.15.1805

[cit0098] Mota Júnior AO, Malavazi I, Soriani FM, Heinekamp T, Jacobsen I, Brakhage AA, Savoldi M, Goldman MHS, da Silva Ferreira ME, Goldman GH. 2008. Molecular characterization of the *Aspergillus fumigatus* NCS-1 homologue, NcsA. Mol Genet Genomics. 280(6):483‐495. doi:10.1007/s00438-008-0381-y. 18830711

[cit0099] Nguyen QB, Kadotani N, Kasahara S, Tosa Y, Mayama S, Nakayashiki H. 2008. Systematic functional analysis of calcium‐signalling proteins in the genome of the rice‐blast fungus, *Magnaporthe oryzae*, using a high‐throughput RNA‐silencing system. Mol Microbiol. 68(6):1348–1365. doi:10.1111/j.1365-2958.2008.06242.x. 18433453

[cit0100] Nierman WC, Pain A, Anderson MJ, Wortman JR, Kim HS, Arroyo J, Berriman M, Abe K, Archer DB, Bermejo C. 2005. Genomic sequence of the pathogenic and allergenic filamentous fungus *Aspergillus fumigatus*. Nature. 438(7071):1151. doi:10.1038/nature04332. 16372009

[cit0101] Odom A, Muir S, Lim E, Toffaletti DL, Perfect J, Heitman J. 1997. Calcineurin is required for virulence of *Cryptococcus neoformans*. Embo J. 16(10):2576–2589. doi:10.1093/emboj/16.10.2576. 9184205 PMC1169869

[cit0102] Okorokova-Facanha AL, Okorokov LA, Ekwall K. 2003. An inventory of the P-type ATPases in the fission yeast *Schizosaccharomyces pombe*. Curr Genet. 43(4):273–280. doi:10.1007/s00294-003-0395-2. 12707717

[cit0103] Otero JM, Vongsangnak W, Asadollahi MA, Olivares-Hernandes R, Maury J, Farinelli L, Barlocher L, Østerås M, Schalk M, Clark A. 2010. Whole genome sequencing of *Saccharomyces cerevisiae*: from genotype to phenotype for improved metabolic engineering applications. BMC Genomics. 11(1):723. doi:10.1186/1471-2164-11-723. 21176163 PMC3022925

[cit0104] Paranjape V, Roy BG, Datta A. 1990. Involvement of calcium, calmodulin and protein phosphorylation in morphogenesis of *Candida albicans*. Microbiol. 136(11):2149–2154. 10.1099/00221287-136-11-2149 2079619

[cit0105] Pittman JK. 2011. Vacuolar Ca^2+^ uptake. Cell Calcium. 50(2):139–146. doi:10.1016/j.ceca.2011.01.004. 21310481

[cit0106] Plattner H, Verkhratsky A. 2013. Ca^2+^ signalling early in evolution–all but primitive. J Cell Sci. 126(10):2141–2150. doi:10.1242/jcs.127449. 23729741

[cit0107] Pongs O, Lindemeier J, Zhu XR, Theil T, Engelkamp D, Krah-Jentgens I, Lambrecht H, Koch KW, Schwemer J, Rivosecchi R, et al. 1993. Frequenin–a novel calcium-binding protein that modulates synaptic efficacy in the drosophila nervous system. Neuron. 11(1):15–28. doi:10.1016/0896-6273(93)90267-u. 8101711

[cit0108] Ponnamperuma C, Sagan C, Mariner R. 1963. Synthesis of adenosine triphosphate under possible primitive earth conditions. Nature. 199(4890):222–226. doi:10.1038/199222a0. 14076678

[cit0109] Prokisch H, Yarden O, Dieminger M, Tropschug M, Barthelmess IB. 1997. Impairment of calcineurin function in *Neurospora crassa* reveals its essential role in hyphal growth, morphology and maintenance of the apical Ca^2+^ gradient. Mol Gen Genet MGG. 256(2):104–114. doi:10.1007/s004380050551. 9349701

[cit0110] Rho H, Jeon J, Lee Y. 2009. Phospholipase C‐mediated calcium signalling is required for fungal development and pathogenicity in *Magnaporthe oryzae*. Mol Plant Pathol. 10(3):337–346. doi:10.1111/j.1364-3703.2009.00536.x. 19400837 PMC6640429

[cit0111] Richmond GS, Smith TK. 2011. Phospholipases A1. Int J Mol Sci. 12(1):588–612. doi:10.3390/ijms12010588. 21340002 PMC3039968

[cit0112] Ries LNA, Rocha MC, de Castro PA, Silva-Rocha R, Silva RN, Freitas FZ, de Assis LJ, Bertolini MC, Malavazi I, Goldman GH. 2017. The *Aspergillus fumigatus* CrzA transcription factor activates chitin synthase gene expression during the caspofungin paradoxical effect. MBio. 8(3):e00705–17. doi:10.1128/mBio.00705-17. 28611248 PMC5472186

[cit0113] Roy BG, Datta A. 1987. A calmodulin inhibitor blocks morphogenesis in *Candida albicans*. FEMS Microbiol Lett. 41(3):327–329. doi:10.1111/j.1574-6968.1987.tb02221.x.

[cit0114] Rusnak F, Mertz P. 2000. Calcineurin: form and function. Physiol Rev. 80(4):1483–1521. doi:10.1152/physrev.2000.80.4.1483. 11015619

[cit0115] Saitoh K, Arie T, Teraoka T, Yamaguchi I, Kamakura T. 2003. Targeted gene disruption of the neuronal calcium sensor 1 homologue in rice blast fungus, *Magnaporthe grisea*. Biosci Biotechnol Biochem. 67(3):651–653. doi:10.1271/bbb.67.651. 12723620

[cit0116] Sánchez-Gracia A, Romero-Pozuelo J, Ferrús A. 2010. Two frequenins in *Drosophila*: unveiling the evolutionary history of an unusual Neuronal Calcium Sensor (NCS) duplication. BMC Evol Biol. 10(1):54. doi:10.1186/1471-2148-10-54. 20170488 PMC2837045

[cit0117] Sanglard D, Ischer F, Marchetti O, Entenza J, Bille J. 2003. Calcineurin A of *Candida albicans*: involvement in antifungal tolerance, cell morphogenesis and virulence. Mol Microbiol. 48(4):959–976. doi:10.1046/j.1365-2958.2003.03495.x. 12753189

[cit0118] Santos M, de Larrinoa IF. 2005. Functional characterization of the *Candida albicans* CRZ1 gene encoding a calcineurin-regulated transcription factor. Curr Genet. 48(2):88–100. doi:10.1007/s00294-005-0003-8. 16044281

[cit0119] Schumacher J, Viaud M, Simon A, Tudzynski B. 2008. The Gα subunit BCG1, the phospholipase C (BcPLC1) and the calcineurin phosphatase co‐ordinately regulate gene expression in the grey mould fungus *Botrytis cinerea*. Mol Microbiol. 67(5):1027–1050. doi:10.1111/j.1365-2958.2008.06105.x. 18208491

[cit0120] Selker EU, Cambareri EB, Jensen BC, Haack KR. 1987. Rearrangement of duplicated DNA in specialized cells of Neurospora. Cell. 51(5):741–752. doi:10.1016/0092-8674(87)90097-3. 2960455

[cit0121] Shemarova IV, Nesterov VP. 2005. Evolution of mechanisms of Ca^2+^-signaling: role of calcium ions in signal transduction in prokaryotes. J Evol Biochem Physiol. 41(1):12–19. doi:10.1007/s10893-005-0029-z. 15810657

[cit0122] Singh SD, Robbins N, Zaas AK, Schell WA, Perfect JR, Cowen LE. 2009. Hsp90 governs echinocandin resistance in the pathogenic yeast *Candida albicans* via calcineurin. PLoS Pathog. 5(7):e1000532. doi:10.1371/journal.ppat.1000532. 19649312 PMC2712069

[cit0123] Song J, Liu X, Zhai P, Huang J, Lu L. 2016. A putative mitochondrial calcium uniporter in *A. fumigatus* contributes to mitochondrial Ca^2+^ homeostasis and stress responses. Fungal Genet Biol. 94:15–22. doi:10.1016/j.fgb.2016.07.001. 27378202

[cit0124] Soriani FM, Malavazi I, da Silva Ferreira ME, Savoldi M, Von Zeska Kress MR, de Souza Goldman MH, Loss O, Bignell E, Goldman GH. 2008. Functional characterization of the *Aspergillus fumigatus* CRZ1 homologue, CrzA. Mol Microbiol. 67(6):1274–1291. doi:10.1111/j.1365-2958.2008.06122.x. 18298443

[cit0125] Stathopoulos AM, Cyert MS. 1997. Calcineurin acts through the CRZ1/TCN1-encoded transcription factor to regulate gene expression in yeast. Genes Dev. 11(24):3432–3444. doi:10.1101/gad.11.24.3432. 9407035 PMC316814

[cit0126] Steinbach WJ, Lamoth F, Juvvadi PR. 2015. Potential microbiological effects of higher dosing of echinocandins. Clin Infect Dis. 61(suppl_6):S669–S677. doi:10.1093/cid/civ725. 26567286

[cit0127] Su L, Ji D, Tao X, Yu L, Wu J, Xia Y. 2017. Recombinant expression, characterization, and application of a phospholipase B from *Fusarium oxysporum*. J Biotechnol. 242:92–100. doi:10.1016/j.jbiotec.2016.12.009. 27940286

[cit0128] Sutton RB, Davletov BA, Berghuis AM, Sudhof TC, Sprang SR. 1995. Structure of the first C2 domain of synaptotagmin I: a novel Ca^2+^/phospholipid-binding fold. Cell. 80(6):929–938. doi:10.1016/0092-8674(95)90296-1. 7697723

[cit0129] Sze H, Liang F, Hwang I, Curran AC, Harper JF. 2000. Diversity and regulation of plant Ca^2+^ pumps: insights from expression in yeast. Annu Rev Plant Biol. 51(1):433–462. doi:10.1146/annurev.arplant.51.1.433. 11543429

[cit0130] Takeda T, Yamamoto M. 1987. Analysis and in vivo disruption of the gene coding for calmodulin in *Schizosaccharomyces pombe*. Proc Natl Acad Sci. 84(11):3580–3584. doi:10.1073/pnas.84.11.3580. 3035538 PMC304918

[cit0131] Tamuli R, Kumar R, Deka R. 2011. Cellular roles of neuronal calcium sensor‐1 and calcium/calmodulin‐dependent kinases in fungi. J Basic Microbiol. 51(2):120–128. doi:10.1002/jobm.201000184. 21077122

[cit0132] Tamuli R, Kumar R, Srivastava DA, Deka R. 2013. Calcium Signalling. In: Kasbekar DP, McCluskey K, editors. Neurospora Geno Mol Biol. First ed. Norfolk: Caister Academic Press; p. 209–226.

[cit0133] Thewes S. 2014. Calcineurin-Crz1 signaling in lower eukaryotes. Eukaryot Cell. 13(6):694–705. doi:10.1128/EC.00038-14. 24681686 PMC4054267

[cit0134] Tisi R, Rigamonti M, Groppi S, Belotti F. 2016. Calcium homeostasis and signaling in fungi and their relevance for pathogenicity of yeasts and filamentous fungi. AIMS Mol Sci. 3(4):505–549. doi:10.3934/molsci.2016.4.505.

[cit0135] Toda T, Shimanuki M, Yanagida M. 1993. Two novel protein kinase C‐related genes of fission yeast are essential for cell viability and implicated in cell shape control. Embo J. 12(5):1987–1995. doi:10.1002/j.1460-2075.1993.tb05848.x. 8491190 PMC413420

[cit0136] Ton V-K, Rao R. 2004. Functional expression of heterologous proteins in yeast: insights into Ca^2+^ signaling and Ca^2+^-transporting ATPases. Am J Physiol Physiol. 287(3):C580–C589. doi:10.1152/ajpcell.00135.2004. 15308463

[cit0137] Tsai H-C, Chung K-R. 2014. Calcineurin phosphatase and phospholipase C are required for developmental and pathological functions in the citrus fungal pathogen *Alternaria alternata*. Microbiol. 160(7):1453–1465. doi:10.1099/mic.0.077818-0. 24763426

[cit0138] Verkhratsky A, Parpura V. 2014. Calcium signalling and calcium channels: evolution and general principles. Eur J Pharmacol. 739:1–3. doi:10.1016/j.ejphar.2013.11.013. 24291103 PMC4037395

[cit0139] Wang JH, Desai R. 1976. A brain protein and its effect on the Ca^2+^-and protein modulator-activated cyclic nucleotide phosphodiesterase. Biochem Biophys Res Commun. 72(3):926–932. doi:10.1016/S0006-291X(76)80220-3. 186066

[cit0140] Williams RJP. 2006. The evolution of calcium biochemistry. Biochim Biophys Acta (BBA)-Mol Cell Res. 1763(11):1139–1146. doi:10.1016/j.bbamcr.2006.08.042. 17023069

[cit0141] Wood V, Gwilliam R, Rajandream M-A, Lyne M, Lyne R, Stewart A, Sgouros J, Peat N, Hayles J, Baker S. 2002. The genome sequence of *Schizosaccharomyces pombe*. Nature. 415(6874):871. doi:10.1038/nature724. 11859360

[cit0142] Xiao G, Ying S-H, Zheng P, Wang Z-L, Zhang S, Xie X-Q, Shang Y, Leger RJS, Zhao G-P, Wang C. 2012. Genomic perspectives on the evolution of fungal entomopathogenicity in *Beauveria bassiana*. Sci Rep. 2(1):483. doi:10.1038/srep00483. 22761991 PMC3387728

[cit0143] Yamamoto T, Takeuchi H, Kanematsu T, Allen V, Yagisawa H, Kikkawa U, Watanabe Y, Nakasima A, Katan M, Hirata M. 1999. Involvement of EF hand motifs in the Ca^2+^‐dependent binding of the pleckstrin homology domain to phosphoinositides. Eur J Biochem. 265(1):481–490. doi:10.1046/j.1432-1327.1999.00786.x. 10491207

[cit0144] York JD, Odom AR, Murphy R, Ives EB, Wente SR. 1999. A phospholipase C-dependent inositol polyphosphate kinase pathway required for efficient messenger RNA export. Sci. 285(5424):96–100. doi:10.1126/science.285.5424.96. 10390371

[cit0145] Yoshida T, Toda T, Yanagida M. 1994. A calcineurin-like gene *ppb1+* in fission yeast: mutant defects in cytokinesis, cell polarity, mating and spindle pole body positioning. J Cell Sci. 107(7):1725–1735. 7983142 10.1242/jcs.107.7.1725

[cit0146] Zelter A, Bencina M, Bowman BJ, Yarden O, Read ND. 2004. A comparative genomic analysis of the calcium signaling machinery in *Neurospora crassa, Magnaporthe grisea*, and *Saccharomyces cerevisiae*. Fungal Genet Biol. 41(9):827–841. doi:10.1016/j.fgb.2004.05.001. 15288019

[cit0147] Zeng W, Mak D-OD, Li Q, Shin DM, Foskett JK, Muallem S. 2003. A new mode of Ca^2+^ signaling by G protein-coupled receptors: gating of IP3 receptor Ca^2+^ release channels by Gβγ. Curr Biol. 13(10):872–876. doi:10.1016/S0960-9822(03)00330-0. 12747838

[cit0148] Zhang H, Zhao Q, Liu K, Zhang Z, Wang Y, Zheng X. 2009. MgCRZ1, a transcription factor of *Magnaporthe grisea*, controls growth, development and is involved in full virulence. FEMS Microbiol Lett. 293(2):160–169. doi:10.1111/j.1574-6968.2009.01524.x. 19260966

